# Multiply Recurrent Episodes of Gastric Emphysema

**DOI:** 10.1155/2011/587198

**Published:** 2011-09-29

**Authors:** Eric M. Pauli, Jonathan M. Tomasko, Vishal Jain, Charles E. Dye, Randy S. Haluck

**Affiliations:** ^1^Division of Minimally Invasive and Bariatric Surgery, Penn State Hershey Milton S. Medical Center and Penn State College of Medicine, Hershey, PA 17033, USA; ^2^Department of Gastroenterology and Hepatology, Penn State Milton S. Hershey Medical Center and Penn State College of Medicine, Hershey, PA 17033, USA

## Abstract

*Introduction*. Gastric emphysema can present both a diagnostic challenge and a life-threatening condition for patients and has only once been reported as being recurrent. *Background*. A 64-year-old male presented with chronic abdominal pain and was found to have gastric pneumatosis on CT scan. The patient was successfully managed conservatively. The cause was attributed to aberrant arterial anatomy and atherosclerosis along with hypotension. The patient has since had 3 episodes of recurrent gastric emphysema, all managed nonoperatively. *Discussion*. To our knowledge, this is the first case of both serial episodes of gastric pneumatosis and gastric mucosal ischemia as a precipitating factor for the development of gastric emphysema.

## 1. Background

First described by Franekel in 1889, gastric emphysema continues to represent an unusual cause of portal venous air in both children and adults [1]. Clinicians must be able to distinguish this benign condition, in which air dissects below the mucosa from a luminal source, from emphysematous gastritis, which is caused by a gas-forming bacterial infection and which has a mortality rate as high as 70% [2, 3]. Generally isolated and self-limited, gastric emphysema has only once been reported to be recurrent [4]. Here, we present the clinical, endoscopic, and radiographic findings in a patient with multiple bouts of gastric emphysema.

**Figure 1:**
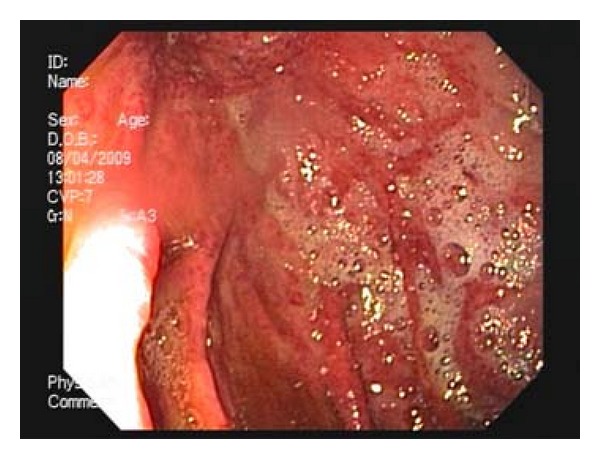
Endoscopic view of the stomach body demonstrating patchy areas of ischemia.

A 64-year-old male with a history of pancreatitis and chronic abdominal pain presented to his community hospital with worsening abdominal pain and hematemesis. Computed tomography (CT) showed diffuse gastric pneumatosis and portal venous air. He was urgently transferred to a tertiary hospital with hypotension and abdominal pain out of proportion to exam findings. Due to a concern for gastric ischemia, he underwent diagnostic laparoscopy; however, his stomach appeared grossly normal. Intraoperative upper endoscopy (EGD) was performed, which showed diffuse edema and mucosal ischemia of the proximal 50% of the stomach (Figure 1).

The patient was managed with proton-pump inhibitor (PPI) therapy and bowel rest. Repeat EGD on hospital day five showed resolution of ischemia. Gastric biopsies would later show no pathologic alteration. The patient's pain resolved and he was subsequently discharged. 

**Figure 2:**
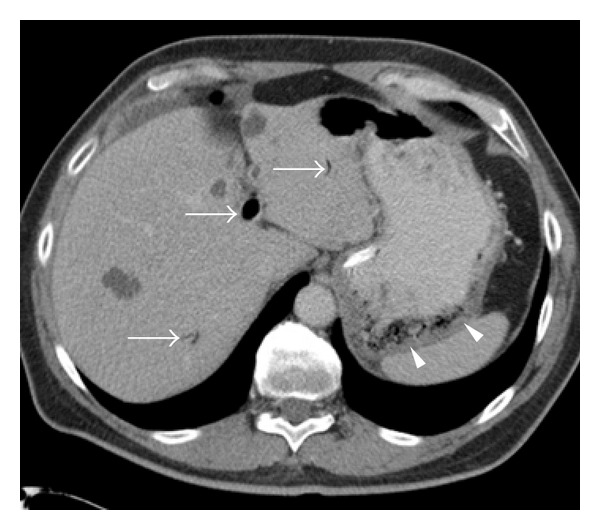
Axial CT scan image demonstrating portal venous gas (arrows) and gastric emphysema (arrowheads). Several benign hepatic cysts are also visualized.

**Figure 3:**
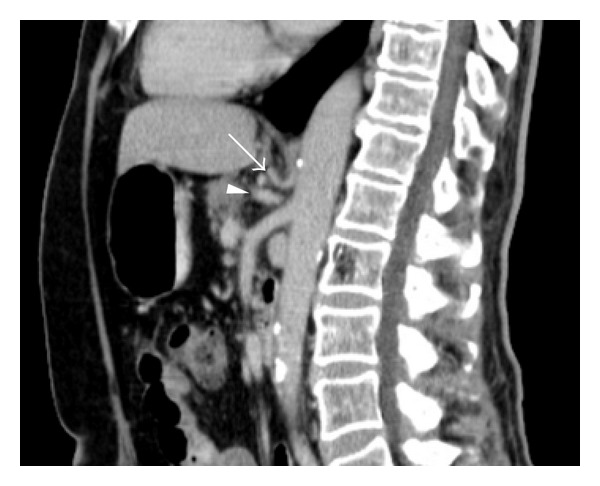
Sagittal CT scan image demonstrating the supra-diaphragmatic origin of the left gastric artery (arrow) and the separate celiac origin (arrowhead). Aortic atherosclerotic calcifications can also be seen.

The patient returned five days later with recurrent abdominal pain, hematemesis, and intermittent episodes of hypotension. Bowel rest and intravenous PPIs were reinstituted. CT scan showed worsening portal venous air and gastric pneumatosis (Figure 2). Repeat EGD demonstrated only mild gastric mucosal ischemia. Mesenteric angiography was performed and showed atherosclerosis and an aberrant left gastric artery, with its origin above the diaphragmatic crus (Figure 3). There was no flow limiting stenosis. He was started on antiplatelet and statin therapy. 

He since has had an additional recurrence of gastric pneumatosis and portal venous air, which was again managed conservatively.

## 2. Discussion

In this case, we suspect that mesenteric atherosclerotic disease, aberrant left gastric arterial anatomy, dyslipidemia, and intermittent bouts of hypotension all contributed to a syndrome of intermittent mesenteric flow insufficiency resulting in mucosal ischemic ulceration, gastric emphysema, and portal vein gas. The patient's pain-induced vomiting may have been a contributing factor in the development of his recurrent episodes of pneumatosis.

Main causes of benign gastric pneumatosis are varied and include gastric outlet obstruction, excessive vomiting, placement of a nasogastric tube, CPR, and ulcer disease [5]. Additional unusual sources of portal venous gas have been described. Zenooz et al. described colonic ischemia as a potential source of portal venous gas, which resolved after colectomy [6]. Blunt abdominal trauma has also been implicated as an uncommon source of gastric pneumatosis, which has been successfully managed nonoperatively with repeat imaging and endoscopy to confirm resolution [7]. Prior to this, aggressive celiotomy and gastric resection had been advocated [8]. 

To our knowledge, this is the first reported case of both serial episodes of gastric pneumatosis (>2) and gastric mucosal ischemia as a precipitating factor for the development of gastric emphysema. Interestingly, nonocclusive mesenteric disease has been associated with the development of portal venous gas in the setting of ischemia [9]. In addition, idiopathic gastric pneumatosis has also been described with a sole presenting symptom of pain by Barbour et al. [10] for which an extensive workup only yielded minor celiac artery artherosclerotic disease as a possible culprit. This patient is currently undergoing evaluation by vascular medicine and vascular surgical services. 

Chronic ischemic gastritis is an unusual entity with a frequently delayed diagnosis, likely from the nonspecific symptoms, inadequate histopathology, and a generalized belief that the stomach has a robust arterial blood supply that protects it from ischemia [11]. This case demonstrates the diligence necessary to make this rare diagnosis and adds ischemic gastritis to the differential diagnosis of gastric emphysema and demonstrates under select circumstances the ability to manage this entity nonoperatively.

##  Disclosure

The authors have no relevant disclosures for the preparation of this paper.

## References

[1] Franekel E (1889). Üeber einen Fall von Gastritis acuta emphysematosa wahrscheinlich mykotishen Ursprungs. *Virchows Archiv*.

[2] Cordum NR, Dixon A, Campbell DR (1997). Gastroduodenal pneumatosis: endoscopic and histological findings. *American Journal of Gastroenterology*.

[3] Taylor DR, Tung JY, Baffa JM, Shaffer SE, Blecker U (2000). Gastric pneumatosis: a case report and review of the literature. *International Pediatrics*.

[4] Kalina M, Rubino M (2009). Recurrent gastric emphysema. *The American Surgeon*.

[5] Nault I, Lauzon C (2007). Gas in the portomesenteric vessels from nonocclusive ischemic bowel disease. *CMAJ*.

[6] Zenooz NA, Robbin MR, Perez V (2007). Gastric pneumatosis following nasogastric tube placement: a case report with literature review. *Emergency Radiology*.

[7] Millward SF, Fortier M (2001). Transient gastric emphysema caused by colonic infraction. *American Journal of Roentgenology*.

[8] Scaglione M, Lassandro F, Pinto F (2001). Gastric pneumatosis and portal vein gas: incidental findings at helical CT after blunt abdominal trauma. *Emergency Radiology*.

[9] Kingsley DD, Albrecht RM, Vogt DM (2000). Gastric pneumatosis and hepatoportal venous gas in blunt trauma: clinical significance in a case report. *The Journal of Trauma*.

[10] Barbour JR, Stokes JP, Uflacker A, Saunders SB, Morgan KA (2010). Spontaneous gastric pneumatosis causing abdominal pain. *The American Surgeon*.

[11] Quentin V, Dib N, Thouveny F, L’Hoste P, Croue A, Boyer J (2006). Chronic ischemic gastritis: case report of a difficult diagnosis and review of the literature. *Endoscopy*.

